# Alternative Care Sites: An Option in Disasters

**DOI:** 10.5811/westjem.2020.4.47552

**Published:** 2020-04-13

**Authors:** Kenneth V. Iserson

**Affiliations:** University of Arizona, Department of Emergency Medicine, Tucson, Arizona

## Abstract

During the current COVID-19 pandemic, the limited surge capacity of the healthcare system is being quickly overwhelmed. Similar scenarios play out when an institution’s systems fail, or when local or regional disasters occur. In these situations, it becomes necessary to use one or more alternative care sites (ACS). Situated in a variety of non-healthcare structures, ACS may be used for ambulatory, acute, subacute, or chronic care. Developing alternative care facilities is the disaster-planning step that moves communities from talking to doing. This commitment pays real dividends if a disaster of any magnitude strikes. This paper discusses the basic criteria for selecting, establishing and ultimately closing an ACS, difficulties of administration, staffing, security, and providing basic supplies and equipment.

## Introduction: Why Have an Alternative Care Site?

During the current COVID-19 pandemic, the limited surge capacity of the healthcare system is being quickly overwhelmed. Similar scenarios play out when an institution’s systems fail—or when local or regional disasters occur. In these situations, it becomes necessary to use one or more alternative care sites (ACS). Situated in a variety of non-healthcare structures, ACS may be used for ambulatory, acute, subacute, or chronic care. Developing alternative care facilities is the disaster-planning step that moves communities from talking to doing. This commitment pays real dividends when a disaster of any magnitude strikes.

Any available site may initially be used to provide healthcare after a disaster. After a massive earthquake in Turkey in 1999, for example, makeshift medical centers were set up on street corners and in ruined buildings.^1^ Five months after Hurricane Katrina, when the media spotlight had dimmed, the New Orleans Convention Center still housed a makeshift medical center. The “emergency rooms” consisted of six military-surplus tents in which about 5000 patients, many of them uninsured poor, were treated each month.^2^

After 9/11, Canadian emergency medicine resident physicians used a New York City high school as a medical facility. They later wrote:

*When we arrived at Stuyvesant High School, we found that there was limited electrical power because many local power grids were knocked out . . . Thus, many of the more intricate procedures, such as suturing, needed to be performed under the illumination of hand-held flashlights. The layout of the triage was very simple. In the main foyer of the school were approximately 8 stretchers clustered together in a makeshift patient care area. Surrounding the cots were dozens of large boxes of unorganized medical equipment, many unopened. The injured arrived either by ambulance or were ambulatory, and promptly triaged. Each stretcher, when possible, was staffed with two nurses, an attending physician, a resident and a medical student. As there were few survivors of the direct disaster, all patients we assessed were emergency response personnel and volunteer rescue workers.*^3^

As the current pandemic enveloped the world, China rapidly built new hospitals, and military units and civilians deployed tent facilities. In the US, states are considering using hotels, ice rinks, stadiums, nursing homes, ships, and recently closed hospitals as alternative care facilities, while the federal government promises that large temporary hospitals will soon be available in the most-affected areas.^4^ Having to do this during a crisis demonstrates our dismal preparation for what is a rare, but predictable, event.

## Issues in Establishing an ACS

Several inter-related issues must be resolved when establishing a viable ACS. These include the following:

- Who will decide to open the facility? Under whose authority will the site be established and run? This should normally be decided at a regional level.
- Who will direct the facility’s operations?
- What types of patients will the facility house and treat? Will the ACS be used to decompress hospitals or nursing homes, or to provide primary care? What patient acuity will it accept? Will it accept oxygen-dependent patients?
- Which available facility will be used for this type of medical care? What will be the selection criteria? How will approval for use be gained? What will be the infrastructure dependencies? Or will the site be a previously designated portable/temporary shelter?
- Who will staff the ACS, including medical support and volunteer staff?
- What durable and disposable supplies and equipment will be available? In what quantities?
- What operational support (meals, sanitation needs, and infrastructure) will be required?
- What policies and what patient documentation will be used?
- Who will decide to close the ACS? What criteria will be used?

## Implementation Difficulties

Planning for ACS is difficult, and takes personnel time, financial resources, and political capital. For these reasons, most regions have abandoned any real ACS planning. Rather, they use a conceptual model, such as “we can use the stadium,” without any further thought. Only when a disaster strikes do they find that the “planning” was woefully inadequate. As a demonstration of how poorly prepared we are, the American Medical Association sent a letter to Congressional leadership on March 19, 2020, demanding funding for frontline treatment: “Providing the funding for the capacity to care for mildly or moderately sick Covid-19 patients in an alternative care site when they cannot appropriately care for themselves at home, such as outpatient facilities or large structures in the community that are in close proximity to a hospital, will provide additional capacity for sicker Covid-19 patients that need more intensive care. This is a crucial step in ensuring we have as much inpatient capacity as possible to respond to the sickest Covid-19 patients.”^5^

In addition to a lack of finances and leadership, another major stumbling block is that, to be effective, multiple groups that traditionally have not done so, must work together.

## Who Controls, Opens, and Closes the Site?

The single most important issue for the successful establishment of an ACS is determining its ownership, command, and control. These are political issues that should be decided at a local or regional (as opposed to institutional) level. Decisions must be made about the individual(s) who can decide whether, when, and where an ACS should be opened, as well as about who has the authority to operate the site.^6^,^7^

Deciding to open an ACS is bound up with bureaucratic and financial implications. While an individual hospital can make the decision alone, the decision will usually be regional, with support from many sources, especially the government

If a hospital decides to open an ACS as part of its emergency operations plan, they assume an enormous burden that few except the largest institutions can manage. This includes the need to find an acceptable facility; to staff and equip it; to establish policies and manage it; to coordinate operations with emergency medical services, the Red Cross, and other community emergency assets; and, lastly, to finance it. While there may be many fewer bureaucratic obstacles to “going it alone,” the sheer magnitude of such an operation presents formidable barriers.

In some cases, where the need is obvious and no leadership has either prepared for or is willing to support establishing an ACS, individual clinicians and support staff may need to open an ACS on their own. That was the case in St. Bernard Parish (county) after Hurricane Katrina, when three family practitioners opened the only medical facility in two parishes in what had been the lobby of the ExxonMobil refinery, located on the highest point in the parish. Using equipment and supplies salvaged from the flood water (with the help of the St. Bernard Paris Sheriff’s Office), they provided care for a week after the storm. They recruited nurses who were willing to stay and help, as well as two family practice residents from a nearby state (one was a relative). Eventually, a disaster medical assistance team (DMAT; AZ-1, from Arizona) assumed responsibility for that ACS.

An exit strategy and exit criteria should be built into the initial plan. The decision to close an ACS is much easier if pre-set and widely understood guidelines control the process. However, ACS closure can be overtaken by events, such as when multiple ACS facilities (opened after Katrina) had to be abandoned when Hurricane Rita threatened the area.

## How Will the ACS be Used?

Most of the decisions about an ACS (staffing, equipment, supplies, and type of structure) flow from the way it will be used. Therefore, that is the first major decision. Possible uses include the following: 6, 7

- Facility in which to quarantine or isolate patients
- Facility to house low-acuity patients from hospitals and nursing homes
- Ambulatory care/vaccination clinic
- Primary triage point to decide where and how patients can best be treated
- Acute care inpatient facility
- Place to provide palliative care for hospitalized patients
- Facility to house patients discharged from hospitals so that they can be released earlier than usual
- Combination of many or all the above functions

Note that many alternative care facilities have multiple functions. Some develop as the situation progresses, although these should not exceed the facility’s structural, staffing, or logistical capabilities.

## Which Type of Facility to Use?

Selecting facilities to serve as an ACS is an imprecise science and varies with the event. Most commonly used are facilities of opportunity, or “buildings of convenience,” which are non-medical structures that can be adapted into an ACS.^6^, ^7^ The building selection process works best if there is first a clear idea of the role planned for the ACS. Buildings typically used as an ACS are listed in Table 1. An often-forgotten fact is that if an ACS can accommodate patients from nursing home/long-term care facilities, those beds can be converted for acute care use (often with an existing oxygen supply).

If there are options when selecting an ACS, especially if it selected in advance of a disaster, some basic questions must be answered to get the best possible facility. Of course, even the best facility will still need much improvisation to make it functional. (Table 2).

## Structural Issues: Selection Criteria

Rate possible alternative care facilities using pre-set criteria (Table 3). Give each criterion a rating (1 to 5) based on how close it comes to the same criterion in a hospital. Sites with the highest total ranking should be the first choice for an ACS. The lack or inadequacy of some criteria may eliminate the structure from consideration, such as the inability to secure the structure or to fulfill most of the criteria listed under “Utilities.”

An example of a rating procedure for each criterion is as follows: 5 = equal to or same as hospital; 4 = similar to that of a hospital, but with some limitations (ie, quantity/condition); 3 = similar to that of a hospital, but with major limitations (ie, quantity/condition); 2 = not similar to that of a hospital and would require minimal modifications to make the facility useable; 1 = not similar to that of a hospital and would require major modifications to make the facility useable; 0 = does not exist in this facility or is not applicable to this event.

Before use begins, facilities’ internal space should be laid out in an organized fashion. A grid system allows clinicians to make “rounds” and know exactly where to find a patient (eg, bed A4). Public health issues are critical (eg, safe food and water supply, sanitation, latrine resources). 6,7

## Staffing and Security

Once a suitable facility is identified, staffing and emergency privileging of healthcare professionals become issues. Staff may be volunteers, off-duty providers from the primary facility, retired clinicians, military personnel, or designated members of disaster response teams (eg, DMAT). Table 4 lists the ideal staffing for each 12-hour shift in a 50-bed inpatient ACS.

Staff members face several issues, including that of arranging for provisions to house and feed the patients. If volunteers are used (as they should be), they should have their own coordinator who understands that some volunteers may not want to do certain tasks (eg, colostomy care, diaper changes). Establish who is going to do what. Note that placing an ACS near a college or university enlarges the potential workforce (eg, sports teams, fraternities, religious groups) to help carry patients, set up equipment, and so forth.^6^,^7^

In chaotic situations, security becomes an extremely important concern, especially since local law enforcement will be stretched thin. Establish a system to identify staff members, patients, and their families. “Planners must develop robust security plans. It is helpful if security personnel have previous experience in dealing with patients, especially those with behavior disorders. The best potential source of security staff would be off-duty hospital security personnel, but these individuals may not be available. Other potential sources would include on- or off-duty police officers, activated members of the National Guard, or volunteers.”^6^ “Security makes patients and staff members feel safe and keeps out troublemakers. Having uniformed people on site (even Reserve Officer Training Corps [ROTC] cadets) makes a real difference.”^6^

Many of these positions are interchangeable. For the ACS to function optimally, everyone must be willing to do any job for which they are qualified.

## Supplies and Equipment

Supplies and equipment for an ACS will vary with its mission and range from extremely primitive to similar to those found in the basic hospital (not including the operating rooms or radiology). Conversely, the lack of specific items, such as oxygen, may limit an ACS’s role. Oxygen and pharmaceuticals are often difficult to obtain during a disaster.

## Oxygen

While generally taken for granted in modern (developed-world) hospitals, oxygen is an expensive, difficult-to-acquire commodity. Table 5 lists the costs, power requirements, and flow rates for some typical portable oxygen delivery systems. In the least-developed countries, the solution is often to use oxygen concentrators, although facility-size units are not commonly available unless purchased in advance. The most common solution is to not accept oxygen-dependent patients in an ACS. Obtaining oxygen from industrial sources may also solve the problem in resource-poor situations. Because industrial-grade oxygen is purer than medical grade, biomedical engineers may be needed to help adapt the connections to medical equipment.^10^ Reducing oxygen use in patients who may not really need it may also be necessary.

## Pharmaceuticals

The nature of the ACS responsibilities (acute and chronic care) and the patient population will determine what pharmaceuticals are needed. In St. Bernard Parish after Hurricane Katrina, for example, the ACS pharmacy (containing items that Sheriff’s officers scrounged from the non-flooded shelves in local pharmacies) was used primarily to supply chronic medications to members of the military and rescue teams. Many medications, despite pharmaceutical company claims and clinician habits, have adequate substitutes that are often readily available. When such needs arise, pharmacists should automatically suggest them for use.^12^ Most ACS will also need to stock medications to provide acute respiratory therapy, acute hemodynamic support, pain control, anxiolytics, antibiotic coverage, and behavioral health maintenance. 6,7

## Alternative Care Site Tools

In March 2020, the US Department of Health and Human Services Office of the Assistant Secretary for Preparedness and Response issued the *Federal Healthcare Resilience Task Force Alternate Care Site (ACS) Toolkit* 2020. It contains a detailed description of the staffing and, most importantly, a checklist to assure that the site is appropriate as an ACS.^13^ Their Healthcare Emergency Preparedness Information Gateway has collected a wide variety of free monographs and teaching tools about ACS (Topic Collection: Alternate Care Sites (including shelter medical care).^14^ The best of these is the California Department of Public Health Government-Authorized Alternate Care Site Training Guide.^7^ In 2009, the Agency for Healthcare Research and Quqlity published three free downloadable resources to help identify and run alternative care sites. One is a monograph, *Disaster Alternate Care Facilities: Selection and Operation*, and two are interactive tools, *Disaster Alternate Care Facility Selection Tool*, and the *Alternate Care Facility Patient Selection Tool*^6^).

## Figures and Tables

**Table 1:** Buildings/structures typically used as alternative care sites during disasters.^6^,^7^

Adult detention facility	Aircraft hanger	Church
Community/recreation center	Convalescent care facility	Fairground
Government building	Hotel/motel	Meeting hall
Military facility	National Guard armory	Same-day surgical center/clinic
School	Shuttered hospital	Sports facility/stadium
Trailer/tent (military or other)		

**Table 2:** Questions to ask when selecting an alternative healthcare site.^8^

Will the structure accommodate the expected number of patients and staff, and the planned activities? Is the structure located in a relatively safe area (culturally and geographically)? Is it structurally sound? Is it easily accessible by ambulance, foot, and automobile/public transportation? Is there adequate electrical power (plus back-up power or the capacity to tie in to large portable generators)? Is there adequate potable water, ventilation, refrigeration, and lighting? Are the ventilation and lighting systems on the back-up generator? Are there also other back-up electrical outlets for critical equipment, such as ventilators? Are there kitchen facilities adequate for the number of people expected (patients, staff, visitors)? Is the entire patient care area wheelchair/stretcher accessible? If elevators are needed, are they on the back-up power system? Will there be separate space for other necessary functions, such as staff sleeping/rest areas, communications center, command center, waiting area, security office, pharmacy, equipment supply and storage areas, chapel/family counseling area, and a morgue? Can the building be secured? Can you control patient and staff traffic? Are there phone and computer access lines? Will cellular phones and radios (two-way, ambulance, public sector, and ham) work within the building without interference? Can lights be dimmed in sleeping and patient care areas? Are the doors > 33 inches wide to permit ambulance stretchers to move through them? Are there areas to load and unload patients and supplies? Ideally, these will accommodate forklifts. Is there parking for patients, staff, and visitors? Are toilet and shower facilities adequate for the anticipated number of patients, staff, and visitors? (*) Does the facility have oxygen or will it be readily available? (*) Is the facility easy to clean for patient use?

(*) Important and often overlooked in planning alternative care sites.

**Table 3:** Criteria to consider in alternative care site selection.

Infrastructure	
Door sizes and stairways adequate for gurneys	Floors
Parking for staff and visitors	Roof
Ventilation	Walls
Toilet facilities/showers (number of)	Loading dock

Total space and layout	

Auxiliary spaces (pharmacy, counselors, chapel)	Staff areas
Lab specimen handling area	Equipment/supply storage area
Mortuary holding area	Family area
Patient decontamination areas	Food supply and prep area
Pharmacy area	Patient care/ward areas

Utilities	

Electrical power (Backup present? Adequate for anticipated equipment?)	Air conditioning (Sufficient for the number of people?)
Lighting	Heating
Refrigeration	Water (Hot?)

Communications	

Communication (number of phones, local/long distance, intercom)	Wired for information technology and Internet access
Two-way radio capability to main facility	Other services

Other factors	

Ability to lock down facility	Laundry
Oxygen delivery capability	Biohazard and other waste disposal
Ownership/other uses during disaster	Proximity to hospitals
Accessibility/proximity to public transportation	Liability insurance coverage

**Table 4:** Idealized staffing for a 50-bed alternative care site per 12-hour shift.^6^,^7^

Physician, 1	Physician Extender (Physician Assistant/Nurse Practitioner), 1	Registered Nurse or Licensed Practical Nurse, 6
Health Technician, 4	Unit Secretary, 2	Respiratory Therapist, 1
Case Manager, 1	Social Worker, 1	Medical Assistant/Phlebotomist, 1
Food Service, 2	Chaplain/Pastoral, 1	Day Care/Pet Care, 1
Volunteer, 4	Engineering/Maintenance, 0.25	Biomedical Engineer, 0.25
Security, 2	Housekeeper, 2	Lab, 1
Patient Transporter, 2		

**Table 5:** Oxygen equipment typically available.^11^

Oxygen generation systems	Oxygen flow rate (L/min)	Power required (kW)	Cost of unit $1000	Oxygen purity (%)
Expeditionary deployable oxygen concentration system	120	8	131	93 ± 3
Portable therapeutic oxygen concentration system	45	7	40	93+
Portable oxygen generation system	33	12	35	93–95
Patient ventilation oxygen concentration system	20	4.3	35	93 ± 3
Home oxygen compressor	3	0.2	2.5	93 ± 3
